# The Potential of circRNA as a Novel Diagnostic Biomarker in Cervical Cancer

**DOI:** 10.1155/2021/5529486

**Published:** 2021-04-07

**Authors:** Shuying Chen, Xiaoyi Yang, Chengxuan Yu, Wenhan Zhou, Qiuyi Xia, Ying Liu, Qiudan Chen, Xiaotong Chen, Yuan Lv, Yong Lin

**Affiliations:** ^1^Department of Laboratory Medicine, Huashan Hospital, Fudan University, Shanghai 200040, China; ^2^School of Medicine, Shanghai Jiao Tong University, Shanghai 200025, China; ^3^Shanghai Medical College, Fudan University, Shanghai 200032, China; ^4^Shanghai University of Medicine & Health Sciences, Shanghai 200237, China; ^5^Department of Central Laboratory, Clnical Laboratory, Jingan District Central Hospital, Fudan University, Shanghai 200040, China

## Abstract

Cervical cancer is the fourth most common cancer among females worldwide. In spite of advances in detection and treatment, it is still one of the most dangerous gynecological malignancies in the world, especially in developing countries, and seriously threatens human health. Circular RNA (circRNA) is a special new type of endogenous noncoding RNA discovered recently. They form a covalently closed continuous loop and are specifically expressed in the eukaryotic transcriptome. With further understanding of circular RNA, a large number of studies have determined the key regulatory role of circRNA in a variety of diseases, especially cancer (including cervical cancer, liver cancer, and lung cancer). In addition, it has also been found that the abnormal expression of circRNA is related to its pathological characteristics in cervical cancer tissue, which can be used as a potential indicator for early screening and diagnosis of cervical cancer, targeted therapy, and prognosis prediction. This article summarizes the recent research achievements of circRNAs in cervical cancer. We briefly described the abnormal expression of circRNA in cervical cancer and discussed the involvement of circRNA in the occurrence process of cervical cancer by regulating cell proliferation, migration, invasion, and apoptosis. We believe that circRNA has potential value as a biomarker in the diagnosis and prognosis of cervical cancer.

## 1. Introduction

### 1.1. Cervical Cancer

In recent years, the incidence and mortality of cervical cancer have remained high. There are approximately 570,000 new cases of cervical cancer and 310,000 deaths due to the disease each year worldwide [1]. Cervical cancer is the fourth most common cancer type diagnosed in females and the fourth most common cause of cancer death in females [2]. The predilection of cervical cancer is young female and often has symptoms such as irregular vaginal bleeding, lower abdominal pain, infertility, and abnormal vaginal secreta. In the past five years, the treatment of recurrent and advanced cervical cancer has improved significantly. However, the average overall survival period of advanced cervical cancer is 16.8 months, and the 5-year overall survival rate of all stages is only 68% [3], which has a great impact and harm on patients' living standard and prognosis.

Early screening of females can reduce the incidence and mortality of cervical cancer. Cervical intraepithelial neoplasia and cervical cancer are mainly closely related to sexually transmitted high-risk human papillomavirus (HPV) infection, which can cause more than 99% of the incidence of cervical cancer [4]. The current screening methods for cervical cancer mainly include cytology and HPV nucleic acid testing [5]. Conventional cytology and liquid-based thin-layer cell testing (TCT) have high sensitivity and specificity for cervical precancerous lesions; although HPV screening and cytology have the same or better results, the specificity of HPV testing is low. It is usually recommended to combine the two methods to improve the accuracy of disease diagnosis. Colposcopy or cervical curettage is used as a histological examination for diagnosis. This method is more accurate when the material is available, but it is more harmful because of the possibility of bleeding, pain, and infection, and its specificity is lower. So colposcopy or cervical curettage cannot be applied in the early screening of cervical cancer.

Therefore, it is particularly important to study the specific molecular mechanisms in the occurrence and development of cervical cancer and to discover new highly sensitive and highly specific noninvasive biomarkers for early screening, diagnosis, treatment, and prognosis monitoring of cervical cancer. At present, the molecular mechanism of circRNAs in cervical cancer is still in the stage of basic research, and it is related to the development of cervical cancer. If further studies can prove that circRNAs can be used as a new biomarker for clinical screening and diagnosis, the harm of histological examination will be avoided. It will greatly optimize diagnosis and targeted therapy. So the clinical application of cervical cancer is particularly important.

## 2. circRNAs

circRNAs are a type of endogenous noncoding RNA, which is widely present in various eukaryotic cells. There is no 5'end cap structure and 3'end polyA tail structure. They form a covalent closed loop different from linear RNA through reverse splicing. RNA molecules are not affected by RNA exonuclease and have certain evolutionary conservation [6].

Based on the molecular characteristics of circRNAs, researchers have discovered that circRNAs have many potential biological functions, including (1) circRNAs can act as sponge molecules for microRNAs (miRNAs), competitively combine with miRNAs, reduce the ability of miRNAs to target mRNA, and participate in different organs of the body regulation of cell physiological functions. (2) circRNAs interact with RNA-binding protein (RBP) to form RNA-protein complex (RPC), which regulates the transcription of linear parent genes. (3) circRNAs form a complex to regulate gene transcription and alternative splicing through RNA-RNA interaction. (4) circRNAs can regulate translation and participate in the coding process of disease-related proteins [7].

miRNAs are believed to act mainly as tumor suppressor genes, and their dysregulation is currently considered to be a common feature of human cancers, and their reduction or inhibition will lead to tumor formation. The decreased expression of mature miRNA may be caused by defects in a certain stage of miRNA generation, such as from base synthesis and uncoupling to exocytosis defects, which ultimately leads to inappropriate expression of oncoprotein miRNA targets. This will lead to stronger proliferation, invasion, and angiogenesis characteristics and affect tissue apoptosis, reduce the degree of differentiation, and ultimately lead to tumor formation [8]. As for the specific mechanism, some studies have pointed out that this reduction is related to epigenetic changes in miRNA gene sequences, such as abnormal DNA methylation and histone modification [8, 9]. In human cancers, the most common cause of tumor suppressor miRNAs deletion is the silencing of the primary transcript by hypermethylation of the CpG island promoter. In cancer, cells undergo global hypomethylation of DNA. This leads to genomic instability and silent transcription of transposable sequences, promoting chromosomal rearrangement and genomic destruction, which are characteristics of tumor cells. However, the CpG island in the promoter region of tumor suppressor genes undergoes DNA hypermethylation, leading to gene silencing and promoting cancer development. Tumor DNA methylation profiles can be used as markers to define tumor type, clinical prognosis, and therapeutic response. miRNAs transcribed from CpG islands undergo DNA methylation-associated suppression, with chromatin similar to coding genes, such as binding of transcriptional repressor methyl-CpG binding domain proteins, and histone modifications associated with silencing, such as acetylation loss of histone H3 and H4. Similar chromatin inhibitory environments have been described for other noncoding RNAs (ncRNAs), such as the ultraconserved regions of transcription, which also undergo promoter CpG island hypermethylation in cancer cells.

In addition to tumor suppressor miRNAs, several well-characterized oncogenic miRNAs (onco-miRNAs) have also been reported in tumors. Interactions between RNA-binding proteins and carcinogenic miRNAs promote the expression of proto-oncogenes or maintain stem cell phenotypes, leading to human cancers [1]. Onco-miRNAs regulate the expression of oncogenic receptors and interfere with cell response to signaling molecules such as transforming growth factor-*β*, Wnt, and Notch.

With the in-depth study of circRNAs, changes in circRNAs not only regulate the relevant physiological functions of the body but also have a broad impact on the biological characteristics of tumors (see Table 1). A large number of studies have confirmed that circRNAs can participate in multiple biological processes such as tumor cell proliferation, invasion, metastasis, and apoptosis as oncogenes or tumor suppressor genes and play important functions in cancer progression. Luan et al. found that the significantly upregulated circRNA_0084043 in melanoma tissue can act as a cavernous body of miR-153-3p and upregulate the expression of Snail, thereby promoting the proliferation, invasion, and migration of melanoma cells and acting as an oncogene in melanoma [21]. Sun et al. found a circRNA (circMYBL2), derived from the cell cycle checkpoint gene MYBL2. Compared with patients with acute myeloid leukemia (AML) without FLT3-ITD mutations, circMYBL2 is expressed higher in AML patients with FLT3-ITD mutations, and its mechanism is mainly increased. The combination of polypyrimidine tract-binding protein 1 (PTBP1) and FLT3 messenger RNA enhances the translation efficiency of FLT3 kinase and participates in the proliferation and differentiation of tumor cells [16]. Feng et al. found that circ-0000190 can affect the proliferation, apoptosis, and cycle of myeloma cells by regulating the miR-767-5p/MAPK4 pathway [15]. In addition, another study showed that circ-ENO1 and its host gene ENO1 are upregulated in lung adenocarcinoma and promote the glycolysis process in lung adenocarcinoma through the miR-22-3p/ENO1 axis and affect tumor growth and metastasis [14].

Based on research in recent years, it can be seen that circRNA promotes or inhibits tumor changes mainly by regulating related signal pathways during tumor cell growth, which has been proven to play a vital regulatory role in a variety of human cancers. However, the function and mechanism of circRNA in cervical cancer are still unclear, and the mechanism of how it acts on specific proteins in the signaling pathway still needs to be lucubrated in depth.

## 3. circRNA and Cervical Cancer

### 3.1. Abnormal Expression

Studies have shown that Arraystar Human circRNA Array technology could be used to identify specific circRNAs that were differentially expressed between one normal cervical epithelial cell line and five cervical cancer cell lines. The technology detected a total of 4760 circRNAs and identified 512 circRNAs with a differential expression of more than 2.0 times between the two groups. It can be seen that the abnormal expression of circRNA is closely related to the occurrence and development of cervical cancer [13]. It is involved in the occurrence and development of cervical cancer through the mutual connection with oncogene-related mRNAs and miRNAs. It can be seen that the abnormal expression of circRNA is closely related to the occurrence and development of cervical cancer.

Song et al. showed through bioinformatics analysis that hsa_circRNA_101996 is highly expressed in cervical cancer tissues compared with matched normal tissues, and its expression level is closely related to the TNM stage, tumor size, lymph node metastasis, and prognosis of cervical cancer patients [12]. This bioinformatics analysis method is helpful to understand the role of circRNA in cervical cancer and lays the foundation for in-depth study of the role of circRNA molecular regulatory network in cervical cancer. In addition, Chen et al. used RT-qPCR to detect the expression of circRNA_0000285 in cervical cancer cells and tissue samples and performed related functional experiments. They found that the expression level of circRNA_0000285 in cervical cancer samples was significantly higher than the corresponding normal tissues. After knocking out circRNA_0000285, the expression of downstream FUS was significantly downregulated, which indicates that circRNA_0000285 may enhance the proliferation and metastasis of cervical cancer by upregulating FUS and provide potential therapeutic targets for the research of cervical cancer [10].

Besides, Cai et al. used western blot and immunohistochemistry methods to confirm that hsa_circ_0000263 can regulate the expression of murine double division 4 (MDM4) by affecting miR-150-5p and ultimately affect the expression of p53 gene. They found that hsa_circ_0000263 significantly upregulated in cervical cancer cells. In addition, knocking down hsa_circ_0000263 can inhibit the proliferation and migration of cervical cancer cells [11].

Yu et al. used high-throughput sequencing to identify 16,893 circRNAs involved in the response of HeLa cells to radiation. Compared with the control group, there were 153 differentially expressed circRNAs, of which 76 were upregulated and 77 were downregulated [17]. At the same time, we used PubMed and Web of Science databases to search related literature studies and found that there are currently few studies on cervical cancer and circRNAs; only hsa_circ_0000745, hsa_circ_0007534, circSLC26A4, circ-AKT1, circCLK3, circRNA8924, circ_0000285, and hsa_circ_0031288 have been reported. The expression in cervical cancer tissues or cell lines is upregulated, and its level is significantly higher than that of adjacent normal tissues or cells, while circRNA_101308 is significantly downregulated in cervical cancer tissues and cells, and its expression is significantly lower than that in normal tissues or cells (see Table 1).

These abnormally expressed circRNAs regulate various genes related to cervical cancer, thereby affecting the biological characteristics of tumors. Their functions are similar to oncogenes and tumor suppressor genes, and they participate in the regulation of tumor cell proliferation, apoptosis, invasion, and migration. Therefore, it is of great significance to study the specific regulatory mechanism of abnormally expressed circRNAs in cervical cancer.

### 3.2. Mechanism of Action

We have found that a variety of circRNAs are abnormally expressed in cervical cancer, and the main mechanism of circRNA's biological function is that circRNA can regulate downstream target genes and their signal pathways by cooperating with miRNA or RBP and other proteins, thereby participating in the cell development of different organs and physiological function adjustment.

Studies have shown that hsa_circRNA_101996 can act as a sponge for miR-8075 and then target TPX2 in cervical cancer cells. At the same time, cell biology methods were used to verify that the upregulation of TPX2 by miR-8075, mediated by has_circRNA_101996, is helpful to the proliferation, migration, and invasion of cervical cancer. It revealed a new mechanism for hsa_circRNA_101996-miR-8075-TPX2 network to promote cervical cancer progression [12]. Chen et al. found that circ_0084927/miR-142-3p/ARL2 can significantly block the proliferation, migration, and invasion of cervical cancer cells and induce cell cycle arrest by western blotting; circ_0084927 targets miR-142-3p, and miR inhibition of -142-3p can reverse the effect of circ_0084927 knockdown. In addition, miR-142-3p can bind to ARL2, and the inhibitory effects of miR-142-3p on cell proliferation, cycle, migration, and invasion are offset by the overexpression of ARL2 [18]. Chen et al. detected by RT-qPCR or western blotting that the ceRNA network of circ_0000285/miR-197-3p/ELK1 can inhibit cell viability and colony formation, block the cell cycle in the G0/G1 phase, and induce cervical cancer cell apoptosis and autophagy, thereby regulating the development of cervical cancer [10]. Accumulated related studies have found that the interaction of circRNA with miRNA or RBP can regulate the expression of target molecules and play an important role in the development of various biological characteristics of cervical cancer.

### 3.3. Potential Markers: Screening Diagnosis

The mechanism of circRNAs affecting the biological function of cervical cancer helps to identify new biological targets. More studies have shown that it exhibits high tissue specificity and good structural stability and makes circRNA resistant to ribonucleic acid. It can be detected in human blood, saliva, urine, and other body fluids, increasing the potential of circRNAs to become noninvasive biomarkers with diagnostic value. circRNA is associated with metastasis in cervical cancer. There are also significant correlations between TNM stage, gender, and age, which makes it more suitable for use as a biomarker for cancer (see Table 1).

Wang et al. found that about 80,000 circRNAs were expressed in cervical tumors and matched normal tissues, of which about 25,000 were expressed in different ways. It is worth noting that many circRNAs detected by this microarray can be verified by RT-qPCR or RNA-seq. However, about 18,000 circRNAs can be reliably detected in cell-free plasma samples, and the expression of about 2700 circRNAs is different after the tumor is surgically removed [19]. The findings of this study confirmed strong evidence for the expression of circRNA in cervical cancer. Huang et al. used RNA sequencing data to identify a set of circRNAs in cervical cancer and matched normal tissues. They found that differentially expressed (DE) circRNAs can distinguish cancer from normal tissues, which indicates that the circRNA expression profile in cervical cancer is comparable to that of normal tissues. There are significant differences [20].

circRNAs are more abundant and stable than other RNAs. With the improvement of related detection technologies such as bioinformatics analysis and next-generation sequencing, the function of circRNA in cervical cancer is becoming more and more widely known, and more circRNAs will be identified as potential new biomarkers for early screening and diagnosis of cervical cancer things.

### 3.4. Prognosis Monitoring

The latest research shows that circRNAs not only affect the proliferation, migration, and invasion of cervical cancer cells by regulating signal pathways but are also related to the pathological parameters of patients with lymph node metastasis and deep muscle infiltration. It also has the function to predict the survival of patients with recurrence and overall survival ability. At present, biomarkers circFoxO3a and circEIF4G2 related to the prognosis of cervical cancer have been found in cervical cancer tissues. Tang et al. used RT-qPCR to detect the expression level of circFoxO3a in the serum of patients with squamous cervical cancer and found that patients with squamous cervical cancer and low serum circFoxO3a expression had a poorer prognosis, which was overall survival and recurrence-free survival. Unfavorable prognostic factors in the period can be used as potential new noninvasive prognostic biomarkers [22]. Mao et al. used RT-qPCR to detect the expression of circEIF4G2 and found that its high expression is closely related to tumor size and lymph node metastasis. In order to check whether the expression of circEIF4G2 can be used as a prognostic indicator for cervical cancer patients, the researchers conducted a related Kaplan–Meier curve analysis and compared cervical cancer samples. It demonstrated that increased expression of circEIF4G2 with cervical cancer samples showed decreased expression of circEIF4G2. The results showed that the higher the expression level of circEIF4G2 in cervical cancer patients, the lower the survival rate of patients. So the increased expression of circEIF4G2 in cervical cancer is closely related to the poor prognosis of patients with cervical cancer [23]. These reports indicate that circRNAs can be used as potential diagnostic and prognostic markers in cervical cancer. The circRNA affecting the prognosis of cervical cancer is shown in Table 1.

### 3.5. Treatment Target

Recently, with the in-depth research on circRNAs, more and more researchers have gained a higher understanding of their expression and function. Abnormally expressed circRNA may become a new specific therapeutic target for different types of cervical cancer. Studies have shown that hsa_circ_0000515 is highly expressed in cervical cancer tissues and cells and plays a tumor-promoting effect, thereby accelerating the proliferation, migration, and invasion of cervical cancer cells [24]. In addition, its downregulation can inhibit cell growth, invasion, inducing apoptosis, and autophagy. Moreover, Yue et al. found that 5 circRNAs (hsa_circRNA_000596, hsa_circRNA_104315, hsa_circRNA_400068, hsa_circRNA_101958, and hsa_circRNA_103519) may compete for endogenous RNA by constructing a regulatory circRNA-micro (mi) RNA-mRNA network, targeting RRM2. These circRNAs are involved in the regulation of mRNA rs5030743 and rs1130609 or other similar SNP treatments of cervical cancer optional chemotherapy drugs [25]. In addition, a recent gene sequencing study showed that the expression of circ_0104541 in cervical tissues was significantly higher than that in paracancerous tissues. In in vitro experiments, downregulating circ_0104541 can significantly inhibit the migration and invasion of cervical cancer cells and can be used as cervical cancer. The potential biomarkers of the disease provide a promising therapeutic target for the targeted therapy of diseases. These findings indicate that circRNAs are promising as new targets for the treatment of cervical cancer [26]. The research summary of the molecular regulatory network of circRNAs in recent years shows that circRNAs have important guiding significance in the screening and diagnosis of cervical cancer, prognostic monitoring, and targeted therapy. However, there are few highly sensitive and highly specific circRNAs that can be used as cervical cancer-related biomarkers. It is still necessary to further study the molecular mechanism of circRNA involvement in cervical cancer and provide new concepts in applications for circRNAs in cancer. See Table 1 for circRNA that can be used as a target for cervical cancer treatment.

## 4. Conclusion and Outlook

By this token, more and more studies have shown that circRNAs are differentially expressed in cervical cancer tissues, and their abnormal expression is related to the clinicopathological characteristics of cervical cancer patients. These circRNAs act as sponge molecules and bind to miRNAs to regulate the expression of downstream target genes and signaling pathways, participate in the biological processes of cervical cancer cell proliferation, invasion, metastasis, cycle, and apoptosis, and influence tumor genesis and development. Considering that it possesses unique advantages, excellent stability, and widespread presence in various body fluids, circRNA is a potential biomarker and important target for screening, diagnosis, treatment, and monitoring of cervical cancer. We believe that the discovery of more and more circRNAs will usher in a new era of gene targeted therapy.

## Figures and Tables

**Table 1:** circRNA expression associated with cervical cancer.

circRNA	Unusual expression	Target genes	Functions	Clinical relevance	Ref.
circRNA_101996	Up	miR-8075	Proliferation, invasion, and migration	TNM stage, tumor size, and lymph node metastasis	[10]
circRNA_0000263	Up	miR-150-5p	Proliferation and migration	Tumor size and lymph node metastasis	[11]
circRNA_0000745	Up	E-cadherin	Proliferation, invasion, and migration	Tumor differentiation and lymphatic invasion	[8]
circRNA_0007534	Up	miR-498	Proliferation and invasion	Tumor size	[9]
circRNA_0031288	Up	hsa-miR-139-3p	Invasion and migration	Lymph node metastasis and infiltration	[12]
circRNA_SLC26A4	Up	miR-1287-5p	Proliferation and invasion	Tumor size	[13]
circRNA_AKT1	Up	miR-942-5p	Proliferation and invasion	Tumor size	[14]
circRNA_CLK3	Up	miR-320a	Proliferation, invasion, and migration	Tumor differentiation, FIGO staging, and stromal infiltration	[15]
circRNA_8924	Up	miR-518d-5p	Proliferation, invasion, and migration	Tumor size, FIGO staging, and muscle invasion	[16]
circRNA__0000285	Up	miR197-3p-ELK1	Cycle and apoptosis	Tumor size	[17]
circRNA_EIF4G2	Up	miR-218	Proliferation, invasion, and migration	Tumor size and lymph node metastasis	[18]
circRNA__0084927	Up	miR-142-3p	Proliferation, invasion, migration, and cycle	Tumor size	[19]
circRNA__FoxO3a	Up	—	Infiltration and migration	Stroma infiltration and lymph node metastasis	[20]
circRNA_101308	Up	miR-26a-5p	Proliferation, invasion, and migration	Lymph node metastasis and deep muscle layer infiltration	[21]

## Data Availability

The data used to support the findings of this study can be obtained by contacting the author at xsjrmt@163.com upon request.
